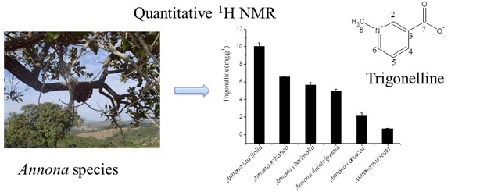# Quantitative analysis of trigonelline in some *Annona* species by proton NMR spectroscopy

**DOI:** 10.1007/s13659-013-0051-6

**Published:** 2013-07-31

**Authors:** Alan Rodrigues Teixeira Machado, Gisele Avelar Lage, Felipe da Silva Medeiros, José Dias de Souza Filho, Lúcia Pinheiro Santos Pimenta

**Affiliations:** Departamento de Química, ICEx, Universidade Federal de Minas Gerais, Belo Horizonte, Minas Gerais, 31270-901 Brasil

**Keywords:** trigonelline, *Annona*, quantitative ^1^H NMR

## Abstract

A quantitative ^1^H NMR method (qHNMR) was used to measure the trigonelline content in the leaves of six species of the *Annona* genus. The methodology employed compared the intensities of the signals at *δ* 9.14 (H-2) and *δ* 0.00, the internal standard TSP-*d*_4_. This measuring method was able to establish the concentration of trigonelline in the range from 0.67 to 10.04 mg·g^−1^ depending on the investigated extract.

## Introduction

Betaines are derivatives amino acids with quaternary nitrogen atom which are commonly found in a variety of living organisms, such as animals, plant, fungi, bacteria and algae[1,2]. Physiologically, these substances have osmotic regulatory properties and can act as a methyl group donor[3]. Among these mesoionic-type substances, trigonelline (Figure 1) is somewhat unique as it displays hypoglycemic, hypocholesterolemic, anti-tumoral and anti-septic properties[4,5]. Moreover, it is well known that trigonelline plays an important role in the resistance process of plants against several pathogens[6].

Trigonelline is widely distributed in mainly herbaceous plants of saline dried habitats[7], and in particular is isolated from leaves of *Trigonella foenum graecum*[8]. In the studies of *Moringa oleifera* performed by Mantur and Kamal[9] using HPLC, different contents (3.55, 2.60, 2.15, 1.90 and 1.60 mg·g$^{-1}$) were found in the pods, leaves, roots, stems and flowers, respectively. Many analytical techniques were employed for the characterization and identification of trigonelline[3,9]. Machado et al.[10] observed by NMR spectroscopy a content of 1.20 mg·g$^{-1}$ of trigonelline in *Coffea Arabica* roots. NMR in particular, is a non-targeted analytical technique where there is no need for previous separations, derivates and even purification with processes being quick and efficient and capable to simultaneously detect a large amount of metabolites in only one analysis[11].

The genus *Annona* has a great commercial importance because it comprises from a large variety of edible fruits. It is noteworthy to mention that the utilization of several species of this genus in popular medicine is due to its diverse pharmacological properties such as its anti-parasitic, anti-ulcer, anti-diarrheal and anti-spasmodic amongst other therapeutic actions[12]. This work herein describes the identification and the quantification of trigonelline in the extracts of various *Annona* species by proton NMR spectroscopy in one and two dimensional experiments.

**Figure 1.** Structure of trigonelline

## Results and Discussion

The proton signals of trigonelline were promptly assigned in all spectra mainly due to their special chemical shifts in the 8.00 to 10.00 ppm region. Figure 2 illustrates one of the various proton NMR spectra obtained for the extracts with the appropriate assignments that were supported by COSY experiments that exhibited the correlations between H-5 to H-4 and H-6.

Generally, the methods described in literature for quantitative analysis by NMR were not performed to determine the longitudinal relaxation time of the trigonelline and TSP-$d_4$ hydrogens. These measurements were crucial to the establishment of the recuperation delay (d1) which were

*To whom correspondence should be addressed. E-mail: alanvisconde@gmail.com

**Figure 2.** Proton NMR spectrum of the *Annona sylvatica* extract leaves (400 MHz, methanol-$d_4$/buffer solution in $D_2O$). The inset shows the expansion from the $^1H$-$^1H$ COSY spectrum.

important to the veracity of the integral of each signal[13]. This delay was set to 5 * longest T1 for the return to the Boltzman equilibrium where all liquid magnetizations were re-established. Therefore, the usual inversion-recovery experiment was applied to determine the T1 values for the hydrogens involved in the quantification (H-2 of trigonelline and methyl group of TSP-$d_4$) (Figure 3). The T1 values of H-2 and methyl hydrogens were 3.06 and 4.05 s, respectively. Thus the value of the recuperation delay (*d1*) was set to 21s to ensure complete relaxation.

**Figure 3.** Longitudinal relaxation times for (a) TSP-$d_4$ and (b) H-2 of trigonelline

Table 1 contains the results of this quantification and it can be seen that *Annona laurifolia* has one with the highest amount of trigonelline (*i.e.* 10.04 mg·g$^{-1}$). This amount of trigonelline can be compared to one of the main natural sources which is *Trigonella foenum graecum* L., where this metabolite is found at the concentration of 13.3 mg·g$^{-1}$[14].This species may be an alternative source of this compound.

In conclusion, the present investigation involved the identification and quantification of trigonelline in leaves of *A. muricata*, *A. laurifolia*, *A. dolabripetala*, *A. sylvatica*, *A. cherimolia* and *A. coriacea*. The efficacy of NMR spectroscopy as an analytical tool has been, once more demonstrated, with its peculiarities, *i.e.*, non-targeted, non-expensive and non-time consuming methodology.

## Experimental Section

**Vegetable Material.** Six species of *Annona* were collected and immediately frozen in liquid nitrogen. Botanical identification was performed by João Renato Stehmann of the Departamento de Botânica do Instituto de Ciências Biológicas of Universidade Federal de Minas Gerais.

**Sample Generic Preparation.** Extracts were prepared according to methodology reported by Kim et al.[15] In the triplicate extractions, 50 mg of leaves were submitted to the extraction by a mixture of methanol-$d_4$ (0.75 mL) and a buffer solution (0.75 mL) of $KH_2PO_4$ in $D_2O$ (90 mM, pH = 6.0) containing TSP-$d_4$ 0.01%. The extraction was conducted by 1 minute vortex stirring followed by 20 minutes sonication and finally, centrifugation. Next, 0.8 mL of the supernatant was transferred to 5 mm NMR tubes.

**NMR Experiments.** NMR experiments were recorded in a Bruker Avance DRX400 (9.4 Tesla) equipped with an inverse multinuclear 5 mm probehead at 303 K. Firstly, the proton longitudinal relaxation times (T1) were determined for the methyl hydrogens of TSP-$d_4$ and H-2 of trigonelline by the inversion-recovery method for optimization of the recuperation delay, d1[13]. The 64k data points proton NMR spectra were

**Table 1. Trigonelline concentrations in the leaves of *Annona* species**

| Plant | Concentration/mg·g$^{-1}$* |
|---|---|
| *Annona muricata* | 0.67 ± 0.04 |
| *Annona laurifolia* | 10.04 ± 0.40 |
| *Annona dolabripetala* | 4.94 ± 0.25 |
| *Annona sylvatica* | 6.56 ± 0.04 |
| *Annona cherimolia* | 5.65 ± 0.27 |
| *Annona coriacea* | 2.11 ± 0.36 |

*mg per gram of dry weight acquired twice for each sample using 16 ppm sweep width. The pulse program 'zgcppr' was the best choice for the water suppression and the 0.3 Hz line broadening were applied for the Fourier transform. The phase and baseline corrections were performed and the TSP-$d_4$ signal calibrated at 0.00 ppm. To ensure the H-2 chemical shift assignment $^1$H NMR spectra besides homonuclear correlation spectroscopy (COSY) were recorded after addition of standard trigonelline to the selected extracts.

## Acknowledgments

We thank FAPEMIG for their financial support and CAPES for the fellowship of ARTM.

**Open Access** This article is distributed under the terms of the Creative Commons Attribution License which permits any use, distribution, and reproduction in any medium, provided the original author(s) and source are credited.
